# Positron Emission Tomography‐Negative Mediastinal Lymph Node Metastasis From Clear Cell Renal Cell Carcinoma 11 Years After Nephrectomy Diagnosed by Endobronchial Ultrasound‐Guided Transbronchial Needle Capillary Sampling

**DOI:** 10.1002/rcr2.70588

**Published:** 2026-04-21

**Authors:** Ryo Hara, Tomoyuki Araya, Takayuki Higashi, Hazuki Takato, Toshiyuki Kita

**Affiliations:** ^1^ Department of Respiratory Medicine NHO Kanazawa Medical Center Kanazawa Japan

**Keywords:** ^18^F‐fluorodeoxyglucose positron emission tomography/computed tomography (^18^F‐FDG PET/CT), clear cell renal cell carcinoma (ccRCC), endobronchial ultrasound‐guided transbronchial needle aspiration (EBUS‐TBNA), endobronchial ultrasound‐guided transbronchial needle capillary sampling (EBUS‐TBNCS), mediastinal lymphadenopathy

## Abstract

A 73‐year‐old man was referred for evaluation of mediastinal lymphadenopathy. He had undergone right nephrectomy for clear cell renal cell carcinoma (ccRCC; pT3aN0M0, stage III) 11 years earlier and had remained recurrence‐free without adjuvant therapy. Positron emission tomography/computed tomography (PET/CT) revealed an enlarged subcarinal lymph node which was non‐FDG avid. Bronchoscopy was performed to obtain a pathological diagnosis. Endobronchial ultrasound‐guided transbronchial needle aspiration (EBUS‐TBNA) initially yielded bloody and inadequate specimens on rapid on‐site cytologic evaluation (ROSE). The sampling technique was therefore switched to endobronchial ultrasound‐guided transbronchial needle capillary sampling (EBUS‐TBNCS), and ROSE demonstrated adequate cellular material with atypical cells. Histopathology and immunohistochemistry confirmed metastatic ccRCC. This case highlights that metastatic ccRCC should be considered in PET‐negative mediastinal lymphadenopathy more than a decade after nephrectomy and that EBUS‐TBNCS may facilitate diagnostic tissue acquisition when conventional EBUS‐TBNA yields bloody specimens.

## Graphical abstract

Metastatic clear cell renal cell carcinoma may present as PET‐negative mediastinal lymphadenopathy even years after nephrectomy. When EBUS‐TBNA yields bloody specimens, switching to EBUS‐TBNCS can facilitate diagnostic tissue acquisition. This approach may improve diagnosis of hypervascular mediastinal lesions.

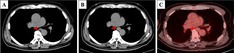

## 1 Introduction

Clear cell renal cell carcinoma (ccRCC) is a hypervascular malignancy that can recur many years after nephrectomy [1]. Metastatic lesions may show variable ^18^F‐fluorodeoxyglucose (^18^F‐FDG) uptake and occasionally present as ^18^F‐FDG positron emission tomography/computed tomography (^18^F‐FDG PET/CT)–negative lymphadenopathy, posing a diagnostic challenge [2]. Endobronchial ultrasound‐guided transbronchial needle aspiration (EBUS‐TBNA) is the standard minimally invasive approach for evaluating mediastinal lymphadenopathy [3]. However, in hypervascular tumours, suction may yield bloody and inadequate specimens. Endobronchial ultrasound‐guided transbronchial needle capillary sampling (EBUS‐TBNCS), a non‐suction technique, may improve tissue acquisition in such situations [4]. We report a rare case of metastatic ccRCC presenting as ^18^F‐FDG PET/CT–negative mediastinal lymphadenopathy 11 years after nephrectomy, in which EBUS‐TBNCS enabled a definitive diagnosis.

## 2 Case Report

**FIGURE 1:**
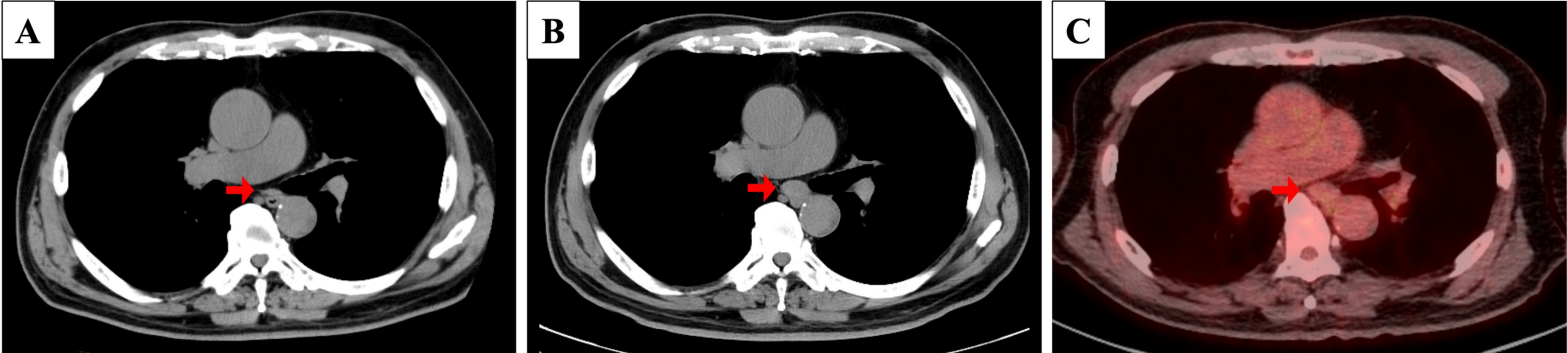
Chest computed tomography (CT) 6 months before showed no mediastinal abnormalities (A). On admission, chest CT revealed subcarinal (station 7) lymphadenopathy (B). ^18^F‐fluorodeoxyglucose positron emission tomography/computed tomography (^18^F‐FDG PET/CT) demonstrated no abnormal ^18^F‐FDG uptake in the station 7 lymph node (C). The station 7 lymph node is indicated by an arrow.

A 73‐year‐old asymptomatic man was referred for evaluation of mediastinal lymphadenopathy. He had undergone right nephrectomy for ccRCC 11 years earlier (pT3aN0M0, stage III) and had remained recurrence‐free without adjuvant therapy. Chest computed tomography (CT) performed six months before referral showed no mediastinal abnormalities (Figure 1A). At presentation, chest and abdominal CT revealed isolated subcarinal (station 7) lymphadenopathy with a short‐axis diameter of 15 mm, without enlargement of other lymph nodes or pulmonary abnormalities (Figure 1B). ^18^F‐FDG PET/CT demonstrated no abnormal FDG uptake in the lesion or elsewhere (Figure 1C).

Bronchoscopy was performed to obtain a pathological diagnosis. On endobronchial ultrasound (EBUS), the station 7 lymph node appeared round with well‐defined margins and heterogeneous echogenicity, and elastography demonstrated a blue‐dominant elastographic image throughout the lymph node. According to the Canadian Lymph Node Score, the lymph node met all four criteria (short‐axis diameter ≥ 10 mm [15 mm in this case], absence of central hilar structure, presence of central necrosis, and well‐defined margins), yielding a total score of 4, suggestive of malignancy [5]. After confirming the absence of vascular structures using Doppler imaging, EBUS‐TBNA was attempted using a 22‐gauge ViziShot 2 needle (Olympus, Tokyo, Japan) with negative pressure applied via a 20‐mL syringe. However, rapid blood backflow occurred after three to‐and‐fro movements, and the obtained specimens were considered inadequate on rapid on‐site cytologic evaluation (ROSE), consisting predominantly of blood with scant cellular material. The sampling technique was therefore switched to EBUS‐TBNCS. The capillary technique was performed as follows: after identification and measurement of the target, a needle was used to puncture the lymph node mass with the stylet in place. Immediately after puncture, the stylet was pushed onto the target to remove the presence of tracheo‐bronchial cells in the tip of the needle. Subsequently, 10 to‐and‐fro movements were performed at each stage, with the stylet withdrawn by one‐third, two‐thirds, and then completely under continuous ultrasonic monitoring [6]. Three passes were performed using this technique. ROSE of the EBUS‐TBNCS specimens demonstrated atypical cells, indicating adequate sampling for diagnosis.

**FIGURE 2:**
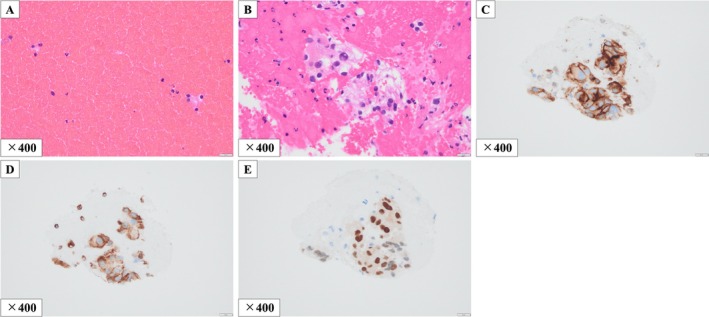
Histopathological findings of the subcarinal (station 7) lymph node. The endobronchial ultrasound‐guided transbronchial needle aspiration specimen consisted predominantly of peripheral blood, with only scattered individual cells exhibiting abundant clear cytoplasm and irregular round‐to‐oval nuclei on haematoxylin and eosin staining (A). In contrast, the endobronchial ultrasound‐guided transbronchial needle capillary sampling specimen revealed atypical cell clusters with a nested architectural pattern on haematoxylin and eosin staining (B). Immunohistochemical staining demonstrated positivity for CD10 (C), vimentin (D), and PAX8 (E) (×400; scale bar = 20 μm).

Histopathological examination showed that the EBUS‐TBNA specimens consisted predominantly of peripheral blood with only scattered atypical cells (Figure 2A), whereas the EBUS‐TBNCS specimens revealed atypical cell clusters with a nested architectural pattern, corresponding to Fuhrman nuclear grade 3 (Figure 2B). Immunohistochemical staining demonstrated positivity for CD10, vimentin, and PAX8, consistent with a diagnosis of metastatic ccRCC (Figure 2C–E). Mediastinal lymph node dissection was subsequently performed with curative intent because the metastasis was solitary and considered oligometastatic disease. The diagnosis of metastatic ccRCC was confirmed histologically. The patient has remained recurrence‐free for 1 year after surgery without additional treatment.

## 3 Discussion

This case illustrates the potential diagnostic value of EBUS‐TBNCS when conventional suction‐based EBUS‐TBNA yields bloody or nondiagnostic specimens. In our patient, suction‐based aspiration resulted in marked blood contamination, rendering the specimens inadequate on ROSE. Although histological examination of the EBUS‐TBNA specimens ultimately allowed a diagnosis, the samples were heavily blood‐contaminated and contained fewer diagnostic cells compared with those obtained using the capillary sampling technique. In contrast, switching to the non‐suction capillary sampling technique at the same target lymph node enabled acquisition of diagnostically adequate tissue, with both ROSE and subsequent histological examination demonstrating sufficient cellularity and reduced blood contamination for a definitive diagnosis of metastatic ccRCC. Previous studies have demonstrated no significant differences between EBUS‐TBNA and EBUS‐TBNCS in terms of adequacy on ROSE and diagnostic yield on histological examination [7, 8]. In addition, studies have shown no significant differences in diagnostic yield among slow‐pull capillary sampling, conventional suction‐based aspiration, and non‐suction capillary sampling techniques [9]; however, non‐suction approaches may reduce blood contamination and facilitate cytological and histological evaluation, particularly in hypervascular lesions. Therefore, when suction‐based EBUS‐TBNA results in bloody aspiration, prompt switching to non‐suction methods may improve specimen quality, thereby facilitating diagnosis.

Another important feature of this case is the late recurrence of ccRCC. Renal cell carcinoma is known to recur long after apparently curative nephrectomy [1]. In the present patient, mediastinal lymph node metastasis developed 11 years after nephrectomy despite a prolonged recurrence‐free interval. This finding underscores the need to consider metastatic disease in patients with a history of ccRCC who present with newly detected lymphadenopathy, even many years after primary treatment.

The case also highlights a limitation of ^18^F‐FDG PET/CT in the evaluation of ccRCC metastases. FDG uptake in ccRCC is variable, and small metastatic lesions may show minimal metabolic activity. Previous studies have demonstrated that the sensitivity of PET/CT increases with lesion size, whereas smaller lesions may yield false‐negative results [2]. Therefore, negative PET/CT findings do not exclude metastatic ccRCC, and histopathological confirmation should be considered when suspicious lymphadenopathy is identified.

In conclusion, metastatic ccRCC should be considered in the differential diagnosis of PET‐negative mediastinal lymphadenopathy even many years after nephrectomy. This case highlights that prompt switching to EBUS‐TBNCS may facilitate diagnostic tissue acquisition, particularly in hypervascular mediastinal lesions.

## Author Contributions

R.H. had full access to all of the data and takes responsibility for the accuracy of the data presentation and writing of the draft of the manuscript. R.H., T.A., T.H., H.T., and T.K. contributed substantially to the case presentation and the review of the manuscript.

## Consent

The authors declare that written informed consent was obtained for the publication of this manuscript and accompanying images and attest that the form used to obtain consent from the patient complies with the Journal requirements as outlined in the author guidelines.

## Conflicts of Interest

The authors declare no conflicts of interest.

## Data Availability

Research data are not shared.

## References

[1] T. L. Rose and W. Y. Kim , “Renal Cell Carcinoma: A Review,” Journal of the American Medical Association 332, no. 12 (2024): 1001–1010. 39196544 10.1001/jama.2024.12848 PMC11790279

[2] N. S. Majhail , J. L. Urbain , J. M. Albani , et al., “F‐18 Fluorodeoxyglucose Positron Emission Tomography in the Evaluation of Distant Metastases From Renal Cell Carcinoma,” Journal of Clinical Oncology 21, no. 21 (2003): 3995–4000. 14581422 10.1200/JCO.2003.04.073

[3] K. Yasufuku , M. Chiyo , Y. Sekine , et al., “Real‐Time Endobronchial Ultrasound‐Guided Transbronchial Needle Aspiration of Mediastinal and Hilar Lymph Nodes,” Chest 126, no. 1 (2004): 122–128. 15249452 10.1378/chest.126.1.122

[4] M. M. Wahidi , F. Herth , K. Yasufuku , et al., “Technical Aspects of Endobronchial Ultrasound‐Guided Transbronchial Needle Aspiration: CHEST Guideline and Expert Panel Report,” Chest 149, no. 3 (2016): 816–835. 26402427 10.1378/chest.15-1216

[5] D. A. Hylton , S. Turner , B. Kidane , et al., “The Canada Lymph Node Score for Prediction of Malignancy in Mediastinal Lymph Nodes During Endobronchial Ultrasound,” Journal of Thoracic and Cardiovascular Surgery 159, no. 6 (2020): 2499–2507. 31926701 10.1016/j.jtcvs.2019.10.205

[6] L. Zuccatosta , F. Mei , M. Sediari , et al., “Diagnostic Accuracy of Slow‐Capillary Endobronchial Ultrasound Needle Aspiration in Determining PD‐L1 Expression in Non‐Small Cell Lung Cancer,” Advances in Respiratory Medicine 91, no. 1 (2023): 1–8. 36648877 10.3390/arm91010001 PMC9844495

[7] R. F. Casal , G. A. Staerkel , D. Ost , et al., “Randomized Clinical Trial of Endobronchial Ultrasound Needle Biopsy With and Without Aspiration,” Chest 142, no. 3 (2012): 568–573. 22156610 10.1378/chest.11-0692 PMC3610596

[8] S. Kassirian , M. A. Mitchell , D. G. McCormack , C. Zeman‐Pocrnich , and I. Dhaliwal , “Rapid on‐Site Evaluation (ROSE) in Capillary Pull Versus Suction Biopsy Technique With Endobronchial Ultrasound‐Transbronchial Needle Aspiration (EBUS‐TBNA),” Journal of Bronchology & Interventional Pulmonology 29, no. 1 (2022): 48–53. 34010221 10.1097/LBR.0000000000000776

[9] Y. Wu , R. Xu , X. Duan , et al., “Comparison of Three Different Puncture Techniques for Endobronchial Ultrasound Transbronchial Needle Aspiration: A Single‐Center, Prospective, Randomized Controlled Study,” BMC Pulmonary Medicine 25, no. 1 (2025): 437. 41029266 10.1186/s12890-025-03917-1 PMC12486861

